# Multi-stakeholder disaster healthcare training as a figurine-based tabletop simulation in coastal Karnataka, India: a kinesthetic teaching–learning experience

**DOI:** 10.1186/s12245-026-01199-w

**Published:** 2026-04-09

**Authors:** Arundhathi Hebbar, Sanjay Kumar Srivastava, Freston Marc Sirur, Aishwarya T. R., Pranav Prakash, Lavanya B. N., Avinash Bhat, Jayaraj Mymbilly Balakrishnan, Manu Sudhi

**Affiliations:** 1 Department of Emergency Medical Technology, Manipal College of Health Professions, Manipal Academy of Higher Education, Manipal, India; 2 National Institute of Advanced Studies, Indian Institute of Science Campus, Bengaluru, India; 3 Centre for Disaster Management, Department of Emergency Medicine, Kasturba Medical College, Manipal Academy of Higher Education, Manipal, India; 4 Department of Hospital Administration, Kasturba Medical College, Manipal Academy of Higher Education, Manipal, India

**Keywords:** Intersectoral collaboration, Disaster preparedness, Simulation training, Tabletop exercise, Health personnel, Hospital administration, Community health workers, Capacity building

## Abstract

**Background:**

Periodic disaster preparedness training is the cornerstone for strengthening coordinated response across multiple tiers of the healthcare system. Integrated multistakeholder training models that are scalable and resource-efficient remain less frequent in low- and middle-income settings.

**Objective:**

To evaluate a structured, stakeholder-specific disaster preparedness training program incorporating figurine-based tabletop simulation as a tactile, kinesthetic learning approach.

**Methods:**

A pre–post educational intervention study was conducted in a tertiary care academic teaching hospital in South India. Seventy-eight participants were enrolled across three stakeholder groups: Emergency Healthcare Professionals (*n* = 26), Hospital Administrators (*n* = 26), and Community Frontline Workers (*n* = 26). The intervention comprised lectures, scenario walkthroughs, and tabletop exercises using miniature figurines to simulate disaster environments. Outcomes were assessed using a structured questionnaire mapped to three domains: Understanding and Awareness; Preparedness and Strategic Planning; and Operational Response and Implementation. Data were analyzed using the Wilcoxon signed-rank test.

**Results:**

All three stakeholder groups demonstrated statistically significant improvements in post-training scores across all domains (*p* < 0.001), with large effect sizes observed for overall score changes.

**Conclusion:**

Figurine-based, kinesthetic tabletop simulation may serve as a scalable and context-adaptable training approach for improving disaster preparedness knowledge, perceived preparedness, and scenario-based decision-making across clinical, administrative, and community stakeholders in resource-limited settings.​​​​​​​​​​​​​​​

**Supplementary Information:**

The online version contains supplementary material available at 10.1186/s12245-026-01199-w.

## Graphical Abstract

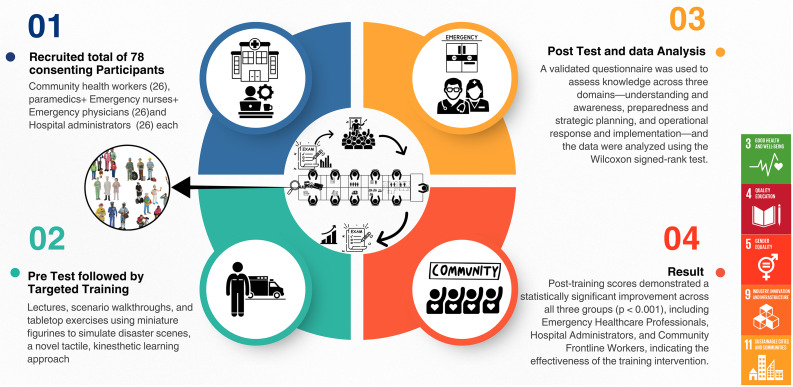

## Introduction

India is highly vulnerable to disasters due to its geo-climatic profile. Nearly 68% of the landmass is drought-prone, 60% is susceptible to earthquakes, over 40 million hectares are flood-affected, and approximately 8% faces cyclone risk. The increasing frequency and severity of these hazards underscore the need for structured preparedness strategies and coordinated multi-stakeholder responses.

Globally, disaster management has transitioned toward preparedness and frameworks for risk-reduction. In India, this shift is reflected in the Disaster Management Act (2005 amended 2025) and the National Disaster Management Policy. However, implementation remains fragmented, under-resourced, and poorly integrated. Evidence evaluating disaster training effectiveness remains limited. Existing literature indicates that although training interventions are associated with improvements in knowledge and perceived competencies, the evidence base is limited by methodological constraints, including reliance on cross-sectional designs, small sample sizes, and insufficient long-term evaluation, thereby restricting causal interpretation [1, 2]. Persistent disparities between prehospital and hospital preparedness further emphasize the need for harmonized, interprofessional training during mass-casualty incidents (MCIs) [3].

Disaster education programs have expanded globally, yet nearly 70% are concentrated in high-income countries, with limited representation from Asia, Africa, and South America [4]. Most interventions emphasize preparedness and response, with minimal focus on mitigation and recovery, reflecting similar fragmentation in India. A scoping review by Emaliyawati et al. (2025) demonstrated that tabletop disaster exercises significantly improve knowledge, teamwork, and confidence among health professionals and students [5]. Although flexible and cost-effective, traditional tabletop formats frequently lack realism and active engagement [5, 6].

Since 1996, WHO-led initiatives have aimed to strengthen hospital managers’ mass -casualty management capabilities, particularly at the district level [7]. However, training demand continues to exceed availability, resulting in preparedness gaps across emergency healthcare systems, hospital administration, and community-based services. Effective disaster preparedness training must therefore extend beyond clinical competencies to include leadership, coordination, and decision-making skills. Simulation-based drills and tabletop exercises have demonstrated effectiveness in reinforcing structured response mechanisms, though scalability and logistical feasibility remain key constraints [5, 6, 8–10]. 

Multi-stakeholder training is essential to ensure synchronized communication and operational coordination during disasters. However, off-site simulations are often constrained by staffing shortages, time limitations, and resource demands. Classroom-based simulations offer economical alternatives, yet scalable and well-documented models remain scarce. Innovative tactile and kinesthetic approaches have been proposed to overcome limitations of passive tabletop formats and enhance engagement, while interprofessional integration across administrative, clinical, and frontline roles remains limited [4–6].

In the prehospital sector, structured training interventions have demonstrated measurable improvements in disaster response. Studies from Kuwait reported low baseline knowledge among providers, with significant post-training gains and a 35% increase in knowledge retention and preparedness among Emergency Medical Technicians (EMTs) and paramedics [11]. Inadequate training has been associated with hesitancy and reduced surge capacity [11]. Emergency Medical Services play a critical role during MCIs and CBRN incidents, yet training often underemphasizes incident-command systems, resource mobilization, and inter-agency coordination [12]. Tabletop simulations have demonstrated effectiveness in improving decision-making and role clarity in these settings [8].

Emergency physicians and nurses play central roles in disaster response. Structured educational interventions have resulted in over 40% improvement in cumulative knowledge and attitude scores among emergency care providers [6, 13]. Nurses often lack disaster exposure in standard curricula, highlighting the need for targeted preparedness training in India [9]. Hospital administrators’ decision-making capacity is equally critical. Mock drills in South India have revealed deficiencies in triage efficiency, staff coordination, and communication, particularly in restriction zones, underscoring the need for standardized assessment tools and continuous training [14].

Community frontline workers frequently serve as first responders during disasters but often lack adequate training and resources. Surveys indicate that over 70% of healthcare professionals are unable to identify early warning signs of floods or droughts, and only 20% have received disaster training in the preceding two years [14]. Evidence from Mysuru further highlights limited foundational emergency-response training among Community Frontline Workers [15].

Persistent barriers, including high simulation costs, limited access to trained educators, and funding constraints, necessitate low-cost, scalable, and sustainable disaster-training solutions [4]. In response to these gaps, the present study evaluates a structured, kinesthetic, figurine-based tabletop simulation model for disaster healthcare training using a pre–post educational assessment framework. While tabletop exercises are widely reported in disaster education literature, published evidence describing tactile, spatially interactive figurine-based simulation formats remains limited. By implementing this model simultaneously across Emergency Healthcare Professionals, Hospital Administrators, and Community Frontline Workers, the study assesses changes in disaster preparedness knowledge, perceived preparedness, and simulated decision-making within an integrated, multi-stakeholder training context. In doing so, it contributes empirical evidence for a structured, resource-efficient disaster preparedness training approach adaptable to low- and middle-income settings.

## Materials and methods

### Study design

A pre–post educational interventional study without a control group was conducted to evaluate the effectiveness of a structured disasterpreparedness training program delivered separately to Emergency Healthcare Professionals, Hospital Administrators, and Community Frontline Workers. Changes in preparedness were assessed using structured pre- and post-training questionnaires across relevant disaster-response domains.

### Study setting and participants

A total of 78 participants were included through purposive sampling, with 26 individuals in each stakeholder group. Sample size was estimated for paired comparisons using the standard formula: *n* = 2 [(Zα + Zβ)² σ² / d²], where Zα = 1.96 corresponding to a 95% confidence level (α = 0.05), and Zβ = 0.84 corresponding to 80% statistical power. Based on prior data, the mean difference between groups (d) was estimated as 0.97, with a pooled standard deviation (σ) of approximately 1.08, corresponding to a large effect size (Cohen’s d ≈ 0.9).

Substituting these values, the calculated sample size was 18 participants per group. Accounting for a 30% potential dropout rate, the final sample size was adjusted to 26 participants per group.

Emergency Healthcare Professionals included physicians, nurses, and paramedics routinely involved in emergency care at a tertiary-care institution in Karnataka, India. Hospital Administrators comprised personnel involved in operations, pharmacy, logistics, and disaster-management coordination. Community Frontline Workers represented local community health volunteers.

Participants were ≥ 18 years of age, actively employed in their respective roles, and able to comprehend training delivered in English or Kannada. Written informed consent was obtained from all participants.

### Intervention

Stakeholder-specific training sessions incorporated structured tabletop exercises (TTEs) using handheld figurines (~ 4 inches) to simulate mass-casualty scenarios within printed hospital floor layouts. The figurines represented patients, responders, administrative personnel, and other operational actors, enabling tactile and spatial engagement in triage prioritization, resource mobilization, crisis communication, surge capacity planning, and activation of the Hospital Emergency Incident Command System (HEICS). This kinesthetic format was designed to facilitate active role-based decision-making and interprofessional coordination within a controlled classroom environment.

**Fig. 1:**
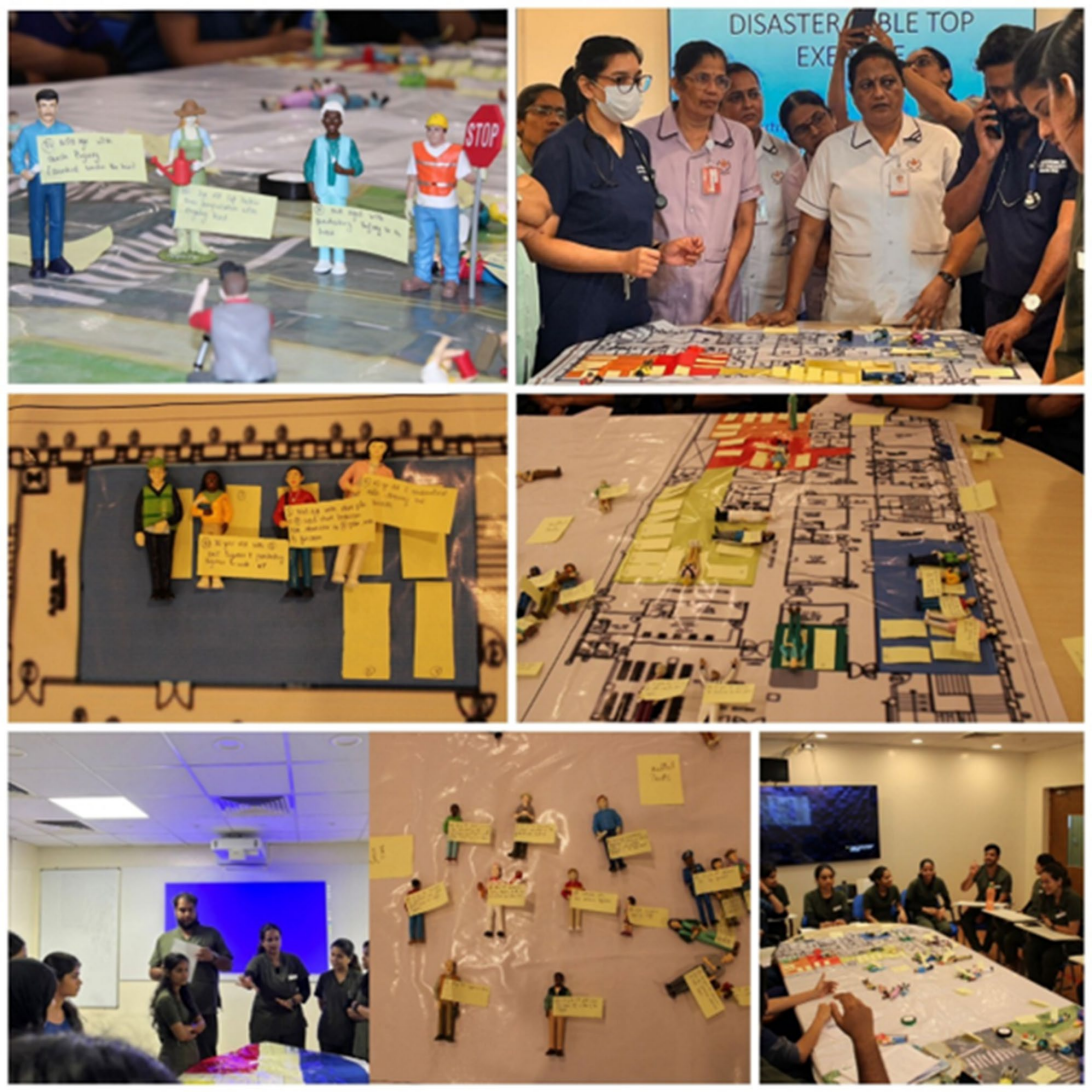
Figurine-based tabletop simulation conducted at the Department of Emergency Medicine, Kasturba Medical College, Manipal, Manipal Academy of Higher Education, Manipal in January 2025, utilizing figurines and floor layouts to simulate and plan mass-casualty response

Sessions were conducted in an academic setting without disrupting clinical operations. Training content was aligned with national disaster-management frameworks and institutional protocols and was adapted to stakeholder roles. Emergency Healthcare Professionals focused on clinical response and coordination; Hospital Administrators on logistics, communication systems, and operational planning; and Community Frontline Workers on early response actions, evacuation procedures, and coordinated communication. An illustrative example of the simulation setup is shown in Fig. 1.

The training content was developed through literature review, institutional experience, and stakeholder needs assessment, and was aligned with national disaster-management frameworks (NDMA, NDMP, DDMA) and relevant DGHS and institutional guidelines. Three role-specific modules were structured accordingly.

The Community Frontline Worker module additionally incorporated the use of the Sachet App for real-time disaster alerts and weather updates to enhance localized preparedness. Sessions were conducted independently for each stakeholder group while maintaining conceptual alignment across shared preparedness domains. The distribution of stakeholder-specific and shared competencies is summarized in Supplementary Table S1.

**Fig. 2:**
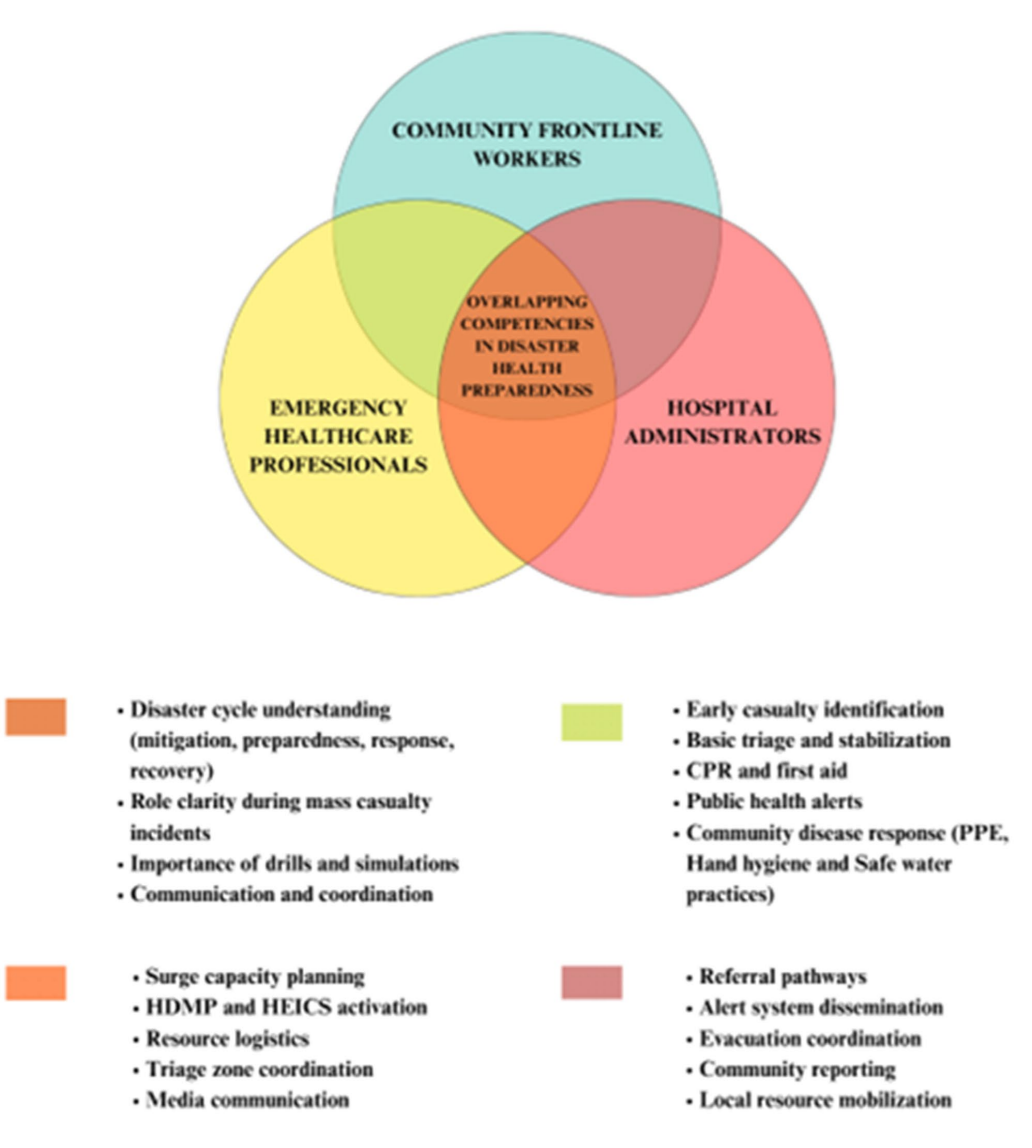
Interpretive module of shared competencies among stakeholder groups in disaster health preparedness. Note: Color-coded overlaps represent shared operational areas between emergency healthcare professionals, hospital administrators, and community frontline workers

An integrated framework emphasizing interprofessional coordination was adopted across modules. Figure 2 illustrates the conceptual overlap of competencies among the three stakeholder groups, highlighting shared domains of triage coordination, communication, evacuation planning, and resource mobilization.

### Data collection and assessment tool

The study was conducted following approval from the Kasturba Hospital Institutional Ethics Committee (IEC Approval No. 196/2024; Approval Date: 30 August 2024; Registration No. ECR/146/Inst/KA/2013/RR-19; DHR Registration No. EC/NEW/INST/2022/KA/0042). The study was registered with the Clinical Trials Registry of India under the registration number CTRI/2024/10/074722 on 04 October 2024.

The assessment questionnaire was developed in alignment with national disaster-management frameworks and relevant institutional guidelines to ensure contextual relevance. The instrument underwent expert content validation only by four subject specialists—two from Emergency Medical Technology, one from Emergency Medicine, and one from Community Medicine—who reviewed the items for relevance, clarity, and appropriateness, and their suggestions were incorporated prior to finalization. Formal psychometric reliability testing and quantitative content validity indexing were not conducted. Therefore, the tool should be interpreted as an exploratory assessment instrument for evaluating disaster preparedness knowledge, perceived preparedness, and scenario-based simulated decision-making as assessed through questionnaire responses.

Questionnaire items were mapped across three analytical domains—Understanding and Awareness; Preparedness and Strategic Planning; and Operational Response and Implementation—aligned with stakeholder responsibilities. Content focus varied by role: Emergency Healthcare Professionals were assessed on hospital response mechanisms and triage principles; Hospital Administrators on logistics, coordination, and strategic planning; and Community Frontline Workers on early warning recognition and basic emergency actions, including use of digital alert systems.

### Measurement

The study assessed three analytical domains—Understanding and Awareness, Preparedness and Strategic Planning, and Operational Response and Implementation—which were designed to mirror the Knowledge, Attitude, and Practice (KAP) framework. This adaptation enabled systematic evaluation of participants’ conceptual understanding, strategic orientation, and applied operational competencies in disaster preparedness and response, while maintaining flexibility across stakeholder groups.

The outcomes assessed in this study reflect changes in disaster preparedness knowledge, perceived preparedness, and simulated decision-making measured through questionnaire-based evaluation.

Data were collected using structured pre- and post-training questionnaires tailored to each stakeholder group but conceptually consistent across domains. For Community Frontline Workers, the tool comprised 16 multiple-choice questions (MCQs) assessing knowledge and practice, and 7 Likert-scale items evaluating attitude. For Hospital Administrators, it included 25 MCQs and 10 Likert-scale items, while for Emergency Healthcare Professionals, it consisted of 25 MCQs and 12 Likert-scale items. Each correct MCQ response was awarded one point, and total knowledge–practice scores were computed by summation. Likert-scale responses were analysed using mean ± standard deviation (SD) to evaluate attitudinal changes.

### Statistical data analysis

All statistical analyses were performed using JAMOVI (version 2.6.25). Descriptive statistics (mean, standard deviation, median, interquartile range, and range) were used to summarize pre- and post-training scores across stakeholder groups. Normality was assessed using the Shapiro–Wilk test. As score distributions were non-normal in two stakeholder groups, the Wilcoxon Signed-Rank Test was applied to compare pre- and post-training scores within each group. Effect sizes (r) were calculated to quantify the magnitude of change.

Effect sizes (r) were calculated from the standardized Wilcoxon test statistic and interpreted using standard thresholds (0.1–0.3 small, 0.3–0.5 moderate, ≥ 0.5 large).

Knowledge scores were additionally categorized as Good (≥ 75%), Average (50–74%), and Poor (< 50%) based on percentage thresholds relative to total possible scores. All participants completed both pre- and post-training assessments, and no data were missing. Statistical significance was defined a priori as *p* < 0.05.

### Bias consideration

Purposive sampling was used to recruit participants across three stakeholder groups, which may introduce selection bias. Anonymized data collection and confidentiality protocols were implemented to reduce response bias; however, the pre–post design remains inherently susceptible to testing effects and social desirability bias, which may influence the magnitude of observed improvements in questionnaire-based outcomes.

### Conceptual framework of the study

The study was structured around a conceptual framework integrating figurine-based, stakeholder-specific tabletop training within a Knowledge–Attitude–Practice (KAP) model, as described in the Methods section. The intervention was grounded in national disaster-management frameworks (NDMA, DDMA) and incorporated stakeholder-specific training across defined analytical domains.

Pre- and post-training assessments were conducted using instruments mapped to three domains—Understanding and Awareness; Preparedness and Strategic Planning; and Operational Response and Implementation—aligned with the KAP framework. The training intervention served as the independent variable, while changes in domain-specific scores were analyzed as dependent outcomes using the Wilcoxon signed-rank test with effect-size estimation.

Table 1 summarizes the alignment between key constructs, instructional strategies, and corresponding assessment methods used in the study.

**Table 1:** Conceptual alignment of KAP constructs with instructional and assessment strategies used in the study

Construct	Operational Focus in Study	Instructional Approach	Assessment Method
Knowledge (Understanding and Awareness)	Understanding of disaster principles, triage systems, HEICS structure, and CBRN readiness	Structured sessions and tabletop simulations reinforcing cognitive learning	Pre–post MCQs assessing conceptual understanding
Attitude (Preparedness and Strategic Planning)	Confidence, preparedness perception, and teamwork orientation during emergencies	Group discussions, reflective debriefing, and role-based participation	Likert-scale items evaluating perceived preparedness and coordination
Practice (Operational Response and Implementation)	Application of disaster principles through decision-making and spatial coordination in simulated settings	Figurine-based tabletop exercises emphasizing triage and coordinated response	Scenario-based MCQs assessing applied operational skills

## Results

A total of 78 participants were enrolled, comprising 26 Emergency Healthcare Professionals, 26 Hospital Administrators, and 26 Community Frontline Workers. All participants completed both pre- and post-training assessments, resulting in complete data availability (100% response rate).

Sociodemographic characteristics are summarized in Table 2. Participants represented diverse professional roles across emergency care, hospital administration, and community health, with professional experience ranging from 2 to 20 years. 

Overall total preparedness scores increased significantly across all stakeholder groups (Wilcoxon signed-rank test, *p* < 0.001)

**Table 2:** Sociodemographic characteristics of study participants (*n* = 78)

Variable	Category	Emergency Healthcare Professionals (*n* = 26)	Hospital Administrators (*n* = 26)	Community Frontline Workers (*n* = 26)
Gender	Male	10 (38.5%)	13 (50%)	—
	Female	16 (61.5%)	13 (50%)	26 (100%)
Age (years)	Range (Mean ± SD)	20–45 (30.0 ± 5.5)	20–40 (30.8 ± 5.7)	25–48 (36.5 ± 6.4)
Years of Professional Experience	Range (Mean ± SD)	2–10 (5.6 ± 2.4)	4–12 (7.8 ± 2.6)	3–20 (9.8 ± 4.2)
Education Qualification		B.Sc./M.Sc. Emergency Medical Technology; (19.2%) M.D. Emergency Medicine (7.7%); B.Sc./GNM Nursing (69.2%)	M.D. Hospital Administration (15.4%); Master of Health Administration (MHA) (26.9%); B Pharm (11.5%); B.Sc. Health Information Management (HIM) (23.1%), Diploma in Basic Sciences / Operations (23.1%)	Higher Secondary Education (PUC / 12th Standard) (57.7%) Secondary Education (10th Standard) (26.9%) B.Sc. Home Science (7.7%) B.A. (7.7%)
Primary Designation / Role	—	5 EMTs (19.2%) 2 MD Emergency Physicians (7.7%) 18 Nurses GNM/BSc (69.2%)	4 MD Hospital Administration (15.4%) 1 Clinical Pharmacist (3.8%) 4 Associate Managers – Operations (15.4%) 1 Deputy MRO (3.8%) 2 Deputy Managers – Operations (7.7%) 2 Senior Executives – Logistics (7.7%) 1 In-charge Logistics (3.8%) 1 Deputy Chief Pharmacist (3.8%) 2 Deputy In-charges – Logistics (7.7%) 1 Fire Safety Officer (3.8%) 1 Assistant Nursing Superintendent (3.8%) 1 In-charge Stationery Department (3.8%) 3 Executives – Ancillary (11.5%) 1 Inventory Department In-charge (3.8%)	26 Community Frontline Workers (100%)

### Framework-aligned domain analysis: understanding, preparedness, and operational response

Results are presented across three analytical domains: Understanding and Awareness; Preparedness and Strategic Planning; and Operational Response and Implementation. For Community Frontline Workers, analysis focused on Knowledge and Practice domains, consistent with their functional roles. Attitudinal outcomes were assessed across all stakeholder groups using Likert-scale measures.

Emergency Healthcare Professionals demonstrated significant improvements across all three analytical domains. The largest proportional gains were observed in incident-command and structured triage components, suggesting strengthened system-level coordination and operational decision-making. Improvements were evident across both conceptual and applied domains. Detailed item-level distributions are presented in Supplementary Table S2.

**Fig. 3:**
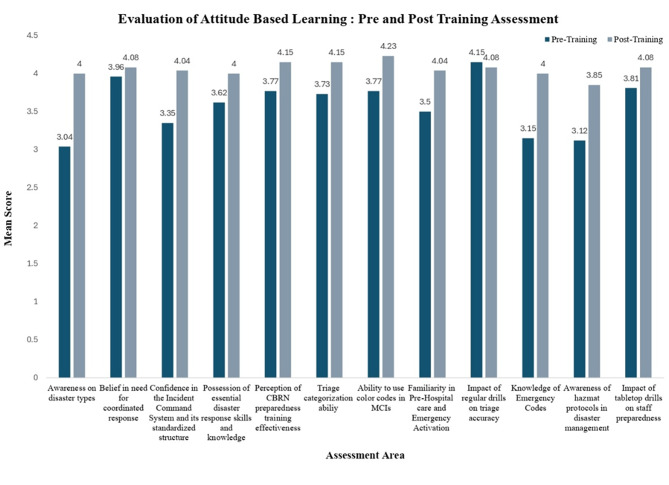
Attitudinal shifts following training – emergency healthcare professional

Post-training assessments indicated attitudinal shifts across several key areas, as captured through Likert scale analysis. Participants demonstrated increased awareness of disaster types and greater confidence in the structure and application of the Hospital Emergency Incident Command System, along with improved familiarity with hazardous material response protocols. Positive changes were also observed in perceptions of coordinated response mechanisms and understanding of emergency codes. A slight decline was noted in one item related to the perceived importance of regular training and drills. These trends are illustrated in Fig. 3.

Correspondingly, post-training reclassification of knowledge levels demonstrated a marked upward shift. Prior to the intervention, 84.6% of participants were categorized as average, 11.5% as poor, and 3.8% as good. Following training, 69.2% were classified as good and 30.8% as average, with the poor category eliminated entirely.

Hospital Administrators demonstrated statistically significant improvements across all three analytical domains following the intervention. Gains were evident in system-level planning constructs, institutional coordination mechanisms, and structured disaster governance processes. Improvements encompassed both conceptual policy understanding and applied operational readiness components. Detailed item-level distributions are presented in Supplementary Table S3.

**Fig. 4:**
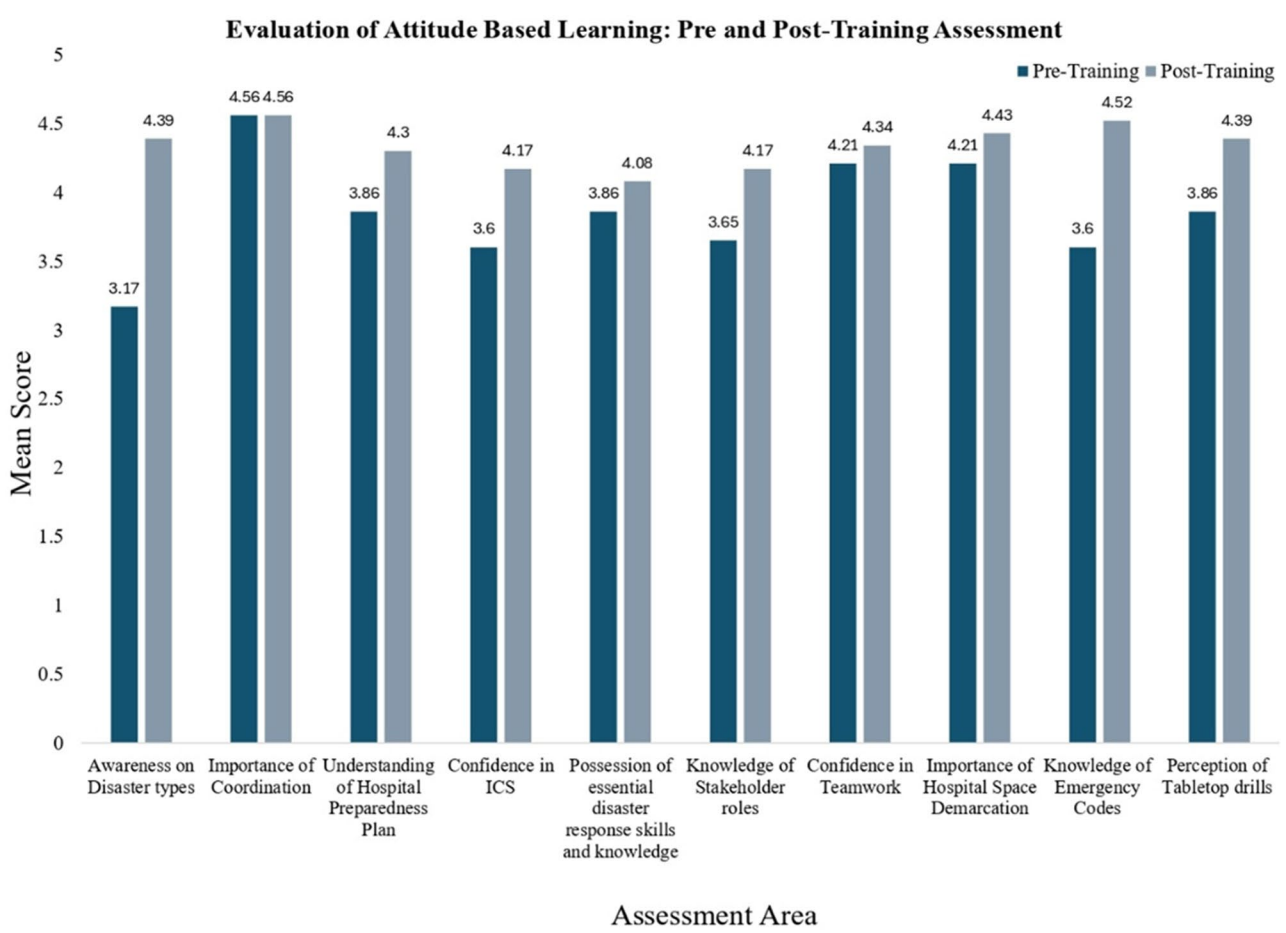
Attitudinal shifts following training – hospital administrators

Post-training assessments demonstrated positive attitudinal shifts across domains, as reflected in Likert scale responses. Increases were observed in perceptions of institutional preparedness, role clarity, structured response alignment, and confidence in simulation-based learning approaches. Perceptions regarding the importance of periodic drills and adequacy of emergency infrastructure remained consistently high across both assessments. These trends are illustrated in Fig. 4.

Overall post-training reclassification of knowledge levels showed a clear upward shift. Prior to the intervention, 46.2% of participants were categorized as average, 23.1% as poor, and 30.8% as good. Following training, 80.8% were classified as good, 19.2% as average, and the poor category was eliminated.

Community Frontline Workers demonstrated significant improvements across both Knowledge and Practice domains following the intervention. Gains were evident in core community preparedness concepts and appropriate field-response actions during disaster scenarios. Improvements reflected strengthened awareness of hazard-specific safety measures and enhanced practical application of emergency response techniques. Detailed item-level distributions are presented in Supplementary Table S4.

**Fig. 5:**
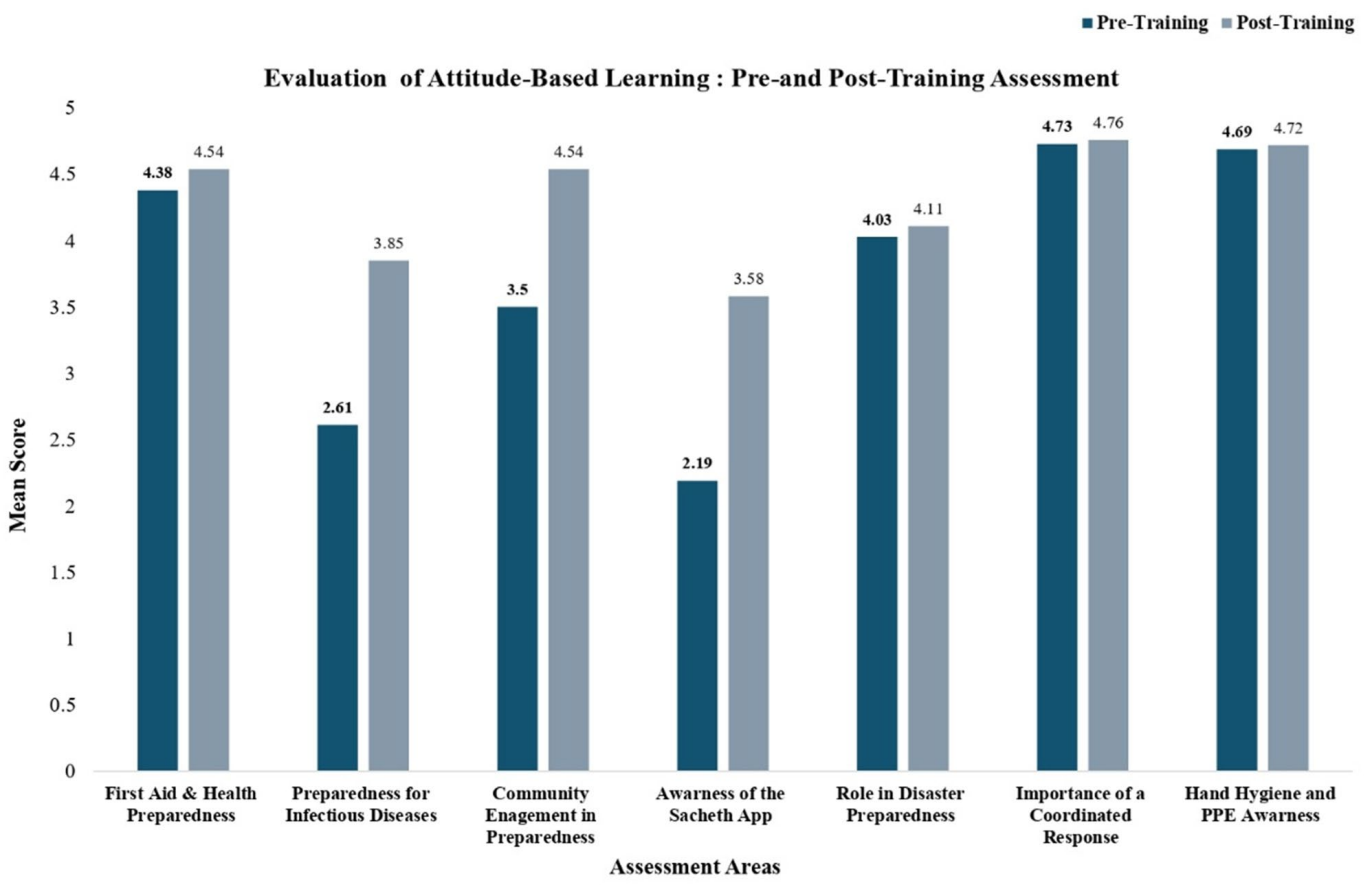
Attitudinal shifts following training – community frontline workers

Attitudinal shifts, as reflected through Likert scale analysis, demonstrated positive post-training changes across domains including preparedness for infectious disease outbreaks, community engagement, and awareness of the Sachet App. Domains with high baseline scores, such as the perceived importance of coordinated response and training, remained consistently elevated following the intervention. These trends are illustrated in Fig. 5.

Detailed item-level pre- and post-training domain outcomes for each stakeholder group—Emergency Healthcare Professionals, Hospital Administrators, and Community Frontline Workers—are presented in Supplementary Tables S2–S4. Table 3 provides a consolidated domain-wise comparison across all groups. Wilcoxon Signed-Rank analysis (Pratt’s method) yielded uniformly high Z-values (≥ 6.17) with *p* < 0.001 across domains. Effect size estimates for overall pre–post score differences were large across stakeholder groups (Hospital Administrators: *r* = 0.84; Community Frontline Workers: *r* = 0.87; Emergency Healthcare Professionals: *r* = 0.87), indicating substantial differences between pre- and post-training scores.

**Table 3:** Domain-wise pre- and post-training total score comparison within stakeholder groups

Group Item group Statistic	Pre Total Score	Post Total Score			Z	*p*-value
**Hospital Administrators**						
Understanding & Awareness						
n	26	26			6.29	< 0.001
Mean (SD)	7 (1.85)	10.38 (1.39)				
Median (Q1, Q3)	7 (6, 8)	10 (9.2, 12)				
Min, Max	4, 11	8, 12				
Preparedness and Strategic Planning						
n	26	26			6.32	< 0.001
Mean (SD)	5.77 (1.84)	6.81 (1.39)				
Median (Q1, Q3)	6 (4.2, 7)	7 (7, 8)				
Min, Max	2, 8	3, 8				
Operational Response and Implementation						
n	26	26			6.32	< 0.001
Mean (SD)	2.69 (0.84)	2.92 (0.48)				
Median (Q1, Q3)	3 (2, 3)	3 (3, 3)				
Min, Max	1, 4	2, 4				
**Community Frontline Workers**						
Knowledge						
n	26	26			6.36	< 0.001
Mean (SD)	6.81 (1.41)	8.35 (0.85)				
Median (Q1, Q3)	7 (6, 8)	9 (8, 9)				
Min, Max	4, 9	6, 9				
Practice						
n	26	26			6.18	< 0.001
Mean (SD)	2.31 (1.09)	4.42 (0.58)				
Median (Q1, Q3)	2 (2, 3)	4 (4, 5)				
Min, Max	0, 4	3, 5				
**Emergency Healthcare Professionals**						
Understanding & Awareness						
n	26	26			6.3	< 0.001
Mean (SD)	4.92 (1.52)	8.54 (1.21)				
Median (Q1, Q3)	5 (4, 6)	8.5 (7.2, 10)				
Min, Max	3, 9	7, 10				
Preparedness and Strategic Planning						
n	26	26			6.3	< 0.001
Mean (SD)	4.08 (1.23)	6.23 (1.63)				
Median (Q1, Q3)	4 (3, 5)	6.5 (5, 7)				
Min, Max	2, 7	3, 9				
Operational Response and Implementation						
n	26	26			6.17	< 0.001
Mean (SD)	2.46 (1.17)	4 (1.26)				
Median (Q1, Q3)	2 (2, 3)	4 (3, 4.8)				
Min, Max	1, 6	2, 6				

n = Number of subjects with observed value; SD = Standard Deviation; Q1 = First Quartile; Q3 = Third Quartile; Min = Minimum; Max = Maximum

p values are based on Wilcoxon Signed Rank test using Pratt’s method

## Discussion

Findings in our study suggest that a structured, stakeholder-specific disaster preparedness intervention incorporating kinesthetic, figurine-based tabletop simulation was associated with improved preparedness-related knowledge and perceived readiness across multiple tiers of the healthcare system in a resource-limited setting. By simultaneously engaging Emergency Healthcare Professionals, Hospital Administrators, and Community Frontline Workers, the model addressed not only individual competency gaps but also the often-fragmented interfaces between institutional and community response structures. Framed within a domain structure aligned to the Knowledge–Attitude–Practice (KAP) model, as described in the Methods section, the intervention enabled structured assessment across domains.

Globally, disaster education programs have expanded over the past two decades, yet important limitations persist. Loke et al. (2021) observed that most interventions focus narrowly on preparedness and response, are concentrated in high-income countries, and rarely integrate multiple tiers of healthcare systems [4]. More recent evidence further indicates that although training interventions improve knowledge and perceived competencies, limitations in evaluation design—such as reliance on self-reported measures, small sample sizes, and lack of long-term follow-up—continue to restrict robust assessment of training effectiveness [1, 2]. The present study addresses these gaps by implementing a structured, multistakeholder intervention within a low-resource Indian context, supported by domain-level assessment and a unified simulation framework adaptable across roles.

Among Emergency Healthcare Professionals, improvements were observed across conceptual, strategic, and operational domains. These findings are consistent with prior studies demonstrating that structured disaster training improves paramedic and emergency care preparedness [11, 13]. However, beyond cognitive gains, this study underscores the added value of tactile, scenario-based simulation in reinforcing structured triage systems, coordinated communication, and incident-command alignment. The observed attitudinal shifts toward greater confidence in system-level coordination further support the argument that experiential learning modalities enhance preparedness beyond knowledge acquisition alone. Prior literature has emphasized that improvements in knowledge do not necessarily translate into operational effectiveness, particularly when training evaluations rely on self-reported outcomes rather than observed performance [1, 2]. The present findings suggest that kinesthetic simulation may help bridge this gap.

For Hospital Administrators, the intervention strengthened clarity in institutional command structures, surge planning, spatial demarcation, and communication roles. Previous assessments of hospital disaster preparedness in India have reported gaps in surge capacity documentation, zone demarcation, and structured command execution during drills [14]. International literature similarly emphasizes the importance of clearly defined command systems and surge protocols in optimizing major incident performance [16, 17]. By embedding administrators within a physically interactive simulation environment, this study operationalized these abstract frameworks into spatially anchored decision-making exercises. This approach appears to have enhanced not only knowledge of governance structures but also confidence in applying them within simulated crisis scenarios.

At the community level, structured inclusion of Community Frontline Workers represents a critical extension of disaster preparedness beyond institutional boundaries. Prior studies from Mysuru and other Indian contexts have documented limited formal disaster training among community-based health workers, including Accredited Social Health Activists (ASHA workers), constraining their response capacity during floods, heatwaves, and infectious outbreaks [15, 18]. More recent evidence further emphasizes that strengthening community-based training and resilience-building initiatives is essential for improving local preparedness and response outcomes [19]. Additionally, structured training of community-level health workers has been shown to enhance disaster response capacity, recovery processes, and overall community resilience [20].

The present intervention demonstrated measurable gains in hazard-specific awareness, emergency response techniques, and digital preparedness, including familiarity with mobile-based alert systems. By contextualizing training within rural realities—such as livestock protection, evacuation challenges, and water safety—the model strengthened locally relevant competencies that are often overlooked in standardized hospital-centric programs. These findings reinforce the need to conceptualize disaster preparedness as a continuum extending from community to tertiary care systems.

The central innovation of this study lies in the use of figurine-based, tactile tabletop simulation as a scalable and resource-efficient pedagogical model. Disaster preparedness education has utilized a range of simulation modalities, each offering different levels of realism, resource requirements, and scalability. Full-scale disaster exercises provide high realism by replicating operational environments with simulated casualties, equipment, and coordinated response activities, enabling practice of triage, inter-agency coordination, and field operations; however, they require extensive logistical planning, trained personnel, and substantial financial resources, limiting routine implementation in resource-constrained settings [10]. Traditional tabletop exercises offer a more feasible alternative by enabling structured discussion of disaster scenarios, resource allocation, and decision-making using maps or prompts in controlled settings; however, their reliance on two-dimensional layouts and verbal narration may limit spatial realism and operational immersion [5, 6, 8–10]. Tabletop simulations have also been applied in disaster nursing education to evaluate competencies related to hazardous-materials incidents and emergency response decision-making, highlighting their value in strengthening situational judgment and critical thinking among nursing personnel [9]. Nurses constitute one of the largest and most critical groups of healthcare professionals involved in disaster preparedness and emergency response, frequently assuming key roles in triage, patient prioritization, coordination of care, and communication during mass-casualty incidents [21]. However, evidence suggests that many nurses report insufficient preparedness for disaster response, emphasizing the need for structured training approaches that strengthen disaster-related competencies [22]. Simulation-based strategies, including tabletop exercises, have been shown to enhance nursing decision-making, situational judgment, and clinical performance in disaster scenarios [9]. In the present study, nursing staff formed a substantial proportion of the Emergency Healthcare Professional participants, further underscoring the relevance of simulation-based preparedness training for this group. Within this context, the figurine-based tabletop simulation used in our study may support the development of competencies directly relevant to emergency nursing practice, particularly in areas such as triage coordination, teamwork, and situational awareness during simulated disaster response.

Similarly, studies involving emergency medical technicians have demonstrated that tabletop drills can enhance understanding of coordination mechanisms, command structures, and role allocation during disaster response compared with traditional field drills [8]. Hospital-based mock drills further emphasize the role of structured simulation exercises in strengthening institutional preparedness, particularly in areas such as incident command, triage organization, and communication systems during mass-casualty incidents [14]. More recently, virtual simulation and virtual-reality training have been introduced to improve immersion and scenario visualization, allowing practice within digitally simulated environments; however, these approaches often require specialized infrastructure, trained facilitators, and significant financial investment, which may limit accessibility in low- and middle-income settings [23]. Similarly, structured low-fidelity simulation models such as the Emergo Train System have demonstrated effectiveness in improving teamwork, triage accuracy, and disaster coordination while maintaining operational feasibility for large-scale training programs [24]. Within this spectrum of simulation modalities, the figurine-based tabletop approach used in the present study extends conventional tabletop exercises by incorporating handheld three-dimensional figurines positioned on realistic layouts. This physically interactive format enables participants to visualize disaster environments, manipulate scenario elements, and simulate operational movements in a spatially structured manner. Such tactile interaction may support improved situational awareness, role clarity, and collaborative decision-making during simulated disaster scenarios while preserving the logistical simplicity and scalability associated with low-fidelity simulation approaches. In low- and middle-income contexts where financial and infrastructural constraints limit advanced simulation capacity, such tactile models offer a pragmatic and economically feasible alternative that bridges the gap between discussion-based tabletop exercises and more resource-intensive high-fidelity disaster simulations. Despite these strengths, several limitations warrant consideration. The study employed a single-center design with purposive sampling, which may limit external generalizability to other geographic or institutional contexts. The pre–post design without a control group introduces potential testing effects and social desirability bias, particularly given participants’ awareness of training objectives. Additionally, the assessment instrument underwent expert content validation only but did not include formal psychometric reliability testing, which may limit the rigor of the assessment tool. The evaluation also focused on immediate post-training outcomes without long-term follow-up to assess retention or real-world behavioral translation. These factors should be considered when interpreting the magnitude of observed improvements.

Nevertheless, the consistent domain-level gains across three distinct stakeholder groups, combined with large effect sizes, suggest that structured, kinesthetic tabletop simulation holds promise as a scalable preparedness strategy. In alignment with the Sendai Framework for Disaster Risk Reduction and WHO emergency preparedness benchmarks, the findings support embedding low-cost, multistakeholder simulation models within routine capacity-building efforts in resource-constrained health systems.

Collectively, this study contributes empirical evidence that disaster preparedness training can move beyond siloed, lecture-based formats toward integrated, tactile, and systems-oriented learning approaches. By bridging cognitive, affective, and operational domains within a unified simulation framework, the figurine-based model offers a practical pathway for strengthening multisectoral disaster readiness in low-resource settings.

## Strengths of the study

A key strength of this study lies in its multistakeholder design, integrating Emergency Healthcare Professionals, Hospital Administrators, and Community Frontline Workers within a unified disaster preparedness framework. This approach enabled assessment of readiness across clinical, administrative, and community tiers of the healthcare system, reflecting real-world response structures. The intervention incorporated structured, stakeholder-specific modules supported by pre- and post-training assessment using a structured, expert content–validated questionnaire and appropriate non-parametric statistical analysis. Training content was aligned with the disaster profile of coastal Karnataka and guided by national disaster management frameworks (NDMA, NIDM, and DDMA), enhancing contextual relevance. The use of handheld figurines within tabletop simulations introduced a tactile, kinesthetic learning modality that facilitated spatial understanding, role clarity, and interactive engagement in a resource-efficient format. Delivery in the local language further improved accessibility while preserving essential technical terminology.

## Limitations

This single-center study using purposive sampling may limit the generalizability of the findings. The pre–post design without a control group introduces potential testing effects and social desirability bias. Outcomes were assessed immediately following the intervention without long-term follow-up to evaluate knowledge retention or behavioral translation into practice. Additionally, although the assessment instrument underwent expert content validation only, formal psychometric reliability testing was not conducted.

## Future directions

Future studies should evaluate this training model across multiple institutions and geographic regions to enhance generalizability. Larger sample sizes and longitudinal follow-up assessments are warranted to determine long-term knowledge retention and potential behavioral translation into practice. Incorporating refresher modules and advanced scenario-based simulations may help sustain engagement and reinforce complex decision-making skills among experienced professionals. Integration of such training into routine healthcare education, orientation programs, and continuing professional development frameworks could further institutionalize disaster preparedness. Expanding the approach to include intersectoral participants, such as police, fire services, and civil defense personnel, may strengthen coordinated emergency response systems. Additionally, qualitative feedback and mixed-methods evaluations could provide deeper insight into participant experiences and contextual adaptation of the training model.

## Conclusion

Our study suggests that a structured disaster preparedness intervention may improve preparedness-related knowledge and perceived readiness across clinical, administrative, and community tiers within a resource-limited setting. By incorporating a tactile learning experience within tabletop simulation, the training supported situational awareness, realism, and context-sensitive decision-making during simulated disaster scenarios. The approach facilitated coordination and role clarity while remaining adaptable to multiple operational contexts. These findings support the potential value of integrating experiential, simulation-based training into routine health-system capacity-building efforts to strengthen disaster preparedness.

## Appendix

**Appendix A:** Parallel presentation of key training components across stakeholder groups

Training Dimension	Emergency Healthcare Professionals	Hospital Administrators	Community Frontline Workers
Foundational Understanding	Risk assessment, hazard identification, vulnerability mapping, disaster cycle, institutional frameworks (NDMA, DDMA) *.	Risk profiling, disaster cycle integration, healthcare system vulnerabilities.	Disaster cycle, personal and community preparedness, public awareness initiatives.
CBRN / Hazard Preparedness	CBRN incidents, triage protocols, burn and blast injury management.	CBRN preparedness integrated within contingency planning.	Response actions for flash floods, lightning, earthquakes, chemical hazards, forest fires, and landslides.
Hospital / System Preparedness	HDMP*, Job Action Cards, HEICS* structure.	HDMP, hospital emergency codes, surge capacity planning.	Introduction to HDMP and HEICS for referral awareness.
Operational Readiness & Response	PPE, pre-hospital care, on-site medical facility planning, resource allocation, surge capacity, CBRN* management	Command and control operations, incident command roles, logistics, and emergency resource allocation, CBRN management	Early warning recognition, disaster kit preparation, evacuation, basic CPR*, hygiene, and PPE*use.
Communication & Coordination	Internal communication, media interface, inter-agency coordination.	Crisis communication, media handling, stakeholder coordination.	Community communication and alert dissemination to authorities.
Digital & Capacity Building	Role-based simulation, use of Sachet App.	Technological integration via Sachet App.	Digital readiness and safety alert dissemination, usage of Sachet App.

* Significance- NDMA: National Disaster Management Authority, DDMA: District Disaster Management Authority, HDMP: Hospital Disaster Management Plan, HEICS: Hospital Emergency Incident Command System; CBRN: Chemical, Biological, Radiological and Nuclear; PPE: Personal Protective Equipment

## Supplementary Information

Below is the link to the electronic supplementary material.

Supplementary Material 1

## Data Availability

The pre- and post-training questionnaires used in this study are available from the corresponding author upon reasonable request.

## Abbreviations

ED: Emergency Medicine Department
EMT: Emergency Medical Technologist
PPE: Personal Protective Equipment
NDMP: National Disaster Management Policy
CBRN: Chemical, Biological, Radiological, and Nuclear
WHO: World Health Organization
MCI: Mass Casualty Incidents
NDMA: National Disaster Management Authority
DDMA: District Disaster Management Authority
EMS: Emergency Medical Services
TTE: Tabletop Exercise
HEICS: Hospital Emergency Incident Command System

## References

[1] Shewchuk S, Wallace J, Seibold M. Evaluations of training programs to improve capacity in K*: a systematic scoping review of methods applied and outcomes assessed. Humanit Soc Sci Commun. 2023;10(1):887. 10.1057/s41599-023-02403-5.

[2] Garavan T, McCarthy A, Sheehan M, Lai Y, Saunders MNK, Clarke N, et al. Measuring the organizational impact of training: The need for greater methodological rigor. Hum Resour Dev Q. 2019;30(3):291–309. 10.1002/hrdq.21345.

[3] Williams J, Nocera M, Casteel C. The Effectiveness of Disaster Training for Health Care Workers: A Systematic Review. Ann Emerg Med. 2008;52(3):211–e2222. 10.1016/j.annemergmed.2007.09.030. 18069087 10.1016/j.annemergmed.2007.09.030

[4] Loke AY, Guo C, Molassiotis A. Development of disaster nursing education and training programs in the past 20 years (2000–2019): A systematic review. Nurse Educ Today. 2021;99:104809. 10.1016/j.nedt.2021.104809. 33611142 10.1016/j.nedt.2021.104809

[5] Emaliyawati E, Ibrahim K, Trisyani Y, Nuraeni A, Sugiharto F, Miladi QN, et al. Enhancing Disaster Preparedness Through Tabletop Disaster Exercises: A Scoping Review of Benefits for Health Workers and Students. Adv Med Educ Pract. 2025;16:1–11. 504705 PubMed PMID: 39807178; PubMed Central PMCID: PMC11725282. 39807178 10.2147/AMEP.S504705 PMC11725282

[6] Sena A, Forde F, Yu C, Sule H, Masters MM. Disaster Preparedness Training for Emergency Medicine Residents Using a Tabletop Exercise. MedEdPORTAL J Teach Learn Resour. 2021;17:11119. 10. 15766/mep_2374-8265.11119 PubMed PMID: 33768151; PubMed Central PMCID: PMC7970644. 10.15766/mep_2374-8265.11119 PMC7970644 33768151

[7] (National Disaster Management Authority (NDMA). Government of India. National Disaster Management Guidelines: Medical Preparedness and Mass Casualty Management. New Delhi: NDMA; 2007.

[8] Chi CH, Chao WH, Chuang CC, Tsai MC, Tsai LM. Emergency medical technicians’ disaster training by tabletop exercise. Am J Emerg Med. 2001;19(5):433–6. 10.1053/ajem.2001.24467. PubMed PMID: 11555806. 11555806 10.1053/ajem.2001.24467

[9] Chiang HH, Ting CW, Chao E, Chen KJ. Using tabletop exercises to evaluate nurses’ clinical performance of hazardous materials disaster management: A cross-sectional study. Nurse Educ Today. 2020;87:104358. 10.1016/j.nedt.2020.104358. PubMed PMID: 32058885. 32058885 10.1016/j.nedt.2020.104358

[10] Alakrawi GA, Al-Wathinani AM, Gómez-Salgado J, Alobaid AM, Abahussian M, Alhazmi R, et al. Evaluating the efficacy of full-scale and tabletop exercises in enhancing paramedic preparedness for external disasters: A quasi-experimental study. Med (Baltim). 2024;103(49):e40777. 10.1097/MD.0000000000040777. PubMed PMID: 39654246; PubMed Central PMCID: PMC11630957. 10.1097/MD.0000000000040777 PMC11630957 39654246

[11] Effect of Mass Casualty Training on Prehospital Care Providers. in Kuwait [Internet]. [cited 2025 Mar 20]. Available from: https://www.researchgate.net/publication/325969873_Effect_of_Mass_Casualty_Training_on_Prehospital_Care_Providers_in_Kuwait.

[12] Beyramijam M, Khankeh HR, Farrokhi M, Ebadi A, Masoumi G, Aminizadeh M. Disaster Preparedness among Emergency Medical Service Providers: A Systematic Review Protocol. Emerg Med Int. 2020;2020:6102940. 10.1155/2020/6102940. PubMed PMID: 33274079; PubMed Central PMCID: PMC7683168. 33274079 10.1155/2020/6102940 PMC7683168

[13] Bhattacharya S, Singh A, Semwal J, Marzo RR, Sharma N, Goyal M, et al. Impact of a training program on disaster preparedness among paramedic students of a tertiary care hospital of North India: A single-group, before-after intervention study. J Educ Health Promot. 2020;9:5. 10.4103/jehp.jehp_423_19. PubMed PMID: 32154300; PubMed Central PMCID: PMC7032020. 32154300 10.4103/jehp.jehp_423_19 PMC7032020

[14] Assessment of disaster preparedness by conducting a mock drill in a tertiary, care teaching, research and referral medical institute in South India [Internet]. [cited 2025 Mar 20]. Available from: https://www.researchgate.net/publication/338337616_ASSESSMENT_OF_DISASTER_PREPAREDNESS_BY_CONDUCTING_A_MOCK_DRILL_IN_A_TERTIARY_CARE_TEACHING_RESEARCH_AND_REFERRAL_MEDICAL_INSTITUTE_IN_SOUTH_INDIA.

[15] Awareness about disaster. management among accredited social health activists in field practice area of a medical college in Mysuru [Internet]. [cited 2025 Mar 20]. Available from: https://www.researchgate.net/publication/337616489_Awareness_about_disaster_management_among_accredited_social_health_activists_in_field_practice_area_of_a_medical_college_in_Mysuru.

[16] Murphy JP, Kurland L, Rådestad M, Rüter A. Hospital incident command groups’ performance during major incident simulations: a prospective observational study. Scand J Trauma Resusc Emerg Med. 2020;28(1):73. 10.1186/s13049-020-00763-4. PubMed PMID: 32727519; PubMed Central PMCID: PMC7389443. 32727519 10.1186/s13049-020-00763-4 PMC7389443

[17] Sheikhbardsiri H, Raeisi AR, Nekoei-Moghadam M, Rezaei F. Surge Capacity of Hospitals in Emergencies and Disasters With a Preparedness Approach: A Systematic Review. Disaster Med Public Health Prep. 2017;11(5):612–20. 10.1017/dmp.2016.178. PubMed PMID: 28264731. 28264731 10.1017/dmp.2016.178

[18] Shannon C. Understanding Community-Level Disaster and Emergency Response Preparedness. Disaster Med Public Health Prep. 2015;9(3):239–44. 10.1017/dmp.2015.28. PubMed PMID: 25826187. 25826187 10.1017/dmp.2015.28

[19] Fu Q, Zhang X. Promoting community resilience through disaster education: Review of community-based interventions with a focus on teacher resilience and well-being. PLoS ONE. 2024;19(1):e0296393. 10.1371/journal.pone.0296393. PubMed PMID: 38166092; PubMed Central PMCID: PMC10760850. 38166092 10.1371/journal.pone.0296393 PMC10760850

[20] Nicholls K, Picou SJ, McCord SC. Training community health workers to enhance disaster resilience. J Public Health Manag Pract JPHMP. 2017;23 Suppl 6 Suppl, Gulf Region Health Outreach Program: S78–84. 10.097/PHH.0000000000000645. PubMed PMID: 28961657. 28961657

[21] Grochtdreis T, de Jong N, Harenberg N, Görres S, Schröder-Bäck P. Nurses’ roles, knowledge and experience in national disaster pre-paredness and emergency response: a literature review. South East Eur J Public Health. 2023 Jan;24. 10.70135/seejph.vi.100.

[22] Aliakbari F, Pirani T, Heidari M, Kheiri S. Effect of operational exercises on nurses’ competence in dealing with disaster. J Educ Health Promot. 2022;11:54. 10.4103/jehp.jehp_429_21. PubMed PMID: 35372603; PubMed Central PMCID: PMC8974978. 35372603 10.4103/jehp.jehp_429_21 PMC8974978

[23] Jung Y. Virtual Reality Simulation for Disaster Preparedness Training in Hospitals: Integrated Review. J Med Internet Res. 2022;24(1):e30600. 10.2196/30600. 35089144 10.2196/30600 PMC8838598

[24] Ratwatte P, Skryabina E, Reedy G, Amlôt R. Benefits of low-fidelity simulations like Emergo Train System (ETS) for healthcare providers emergency preparedness: a scoping review study. J Public Health Emerg. 2025;9(0). 10.21037/jphe-24-55.

