# Supplementary material for: Multi-stakeholder disaster healthcare training as a figurine-based tabletop simulation in coastal Karnataka, India: a kinesthetic teaching–learning experience
Source: Int J Emerg Med. 2026 Apr 9;19:91. doi: 10.1186/s12245-026-01199-w (PMC13063577; doi:10.1186/s12245-026-01199-w)
Supplement: Supplementary file 1 — Supplementary Material 1 [file 12245_2026_1199_MOESM1_ESM.docx]

**Table S1** - Parallel Presentation of Key Training Components Across Stakeholder Groups

| Training Dimension | Emergency Healthcare Professionals | Hospital Administrators | Community Frontline Workers |
| --- | --- | --- | --- |
| Foundational Understanding | Risk assessment, hazard identification, vulnerability mapping, disaster cycle, institutional frameworks (NDMA, DDMA) *. | Risk profiling, disaster cycle integration, healthcare system vulnerabilities. | Disaster cycle, personal and community preparedness, public awareness initiatives. |
| CBRN / Hazard Preparedness | CBRN incidents, triage protocols, burn and blast injury management. | CBRN preparedness integrated within contingency planning. | Response actions for flash floods, lightning, earthquakes, chemical hazards, forest fires, and landslides. |
| Hospital / System Preparedness | HDMP*, Job Action Cards, HEICS* structure. | HDMP, hospital emergency codes, surge capacity planning. | Introduction to HDMP and HEICS for referral awareness. |
| Operational Readiness & Response | PPE, pre-hospital care, on-site medical facility planning, resource allocation, surge capacity, CBRN* management | Command and control operations, incident command roles, logistics, and emergency resource allocation, CBRN management | Early warning recognition, disaster kit preparation, evacuation, basic CPR*, hygiene, and PPE*use. |
| Communication & Coordination | Internal communication, media interface, inter-agency coordination. | Crisis communication, media handling, stakeholder coordination. | Community communication and alert dissemination to authorities. |
| Digital & Capacity Building | Role-based simulation, use of Sachet App. | Technological integration via Sachet App. | Digital readiness and safety alert dissemination, usage of Sachet App. |

* *Significance- NDMA: National Disaster Management Authority, DDMA: District Disaster Management Authority, HDMP: Hospital Disaster Management Plan, HEICS: Hospital Emergency Incident Command System; CBRN: Chemical, Biological, Radiological and Nuclear; PPE: Personal Protective Equipment*

**Table S2**: *Comparison of Pre- and Post-Training Knowledge and Practice Scores among Emergency Healthcare Professionals*

| Questions | Correct Answers | |
| --- | --- | --- |
|  | Pre-test *n* (%) | Post-test *n* (%) |
| 1. Understanding and Awareness |  |  |
| Primary response organization in India | 24 (92.3) | 26 (100.0) |
| Type of disaster requiring strategic patient influx management. | 21 (80.8) | 25 (96.2) |
| Function of triage in mass casualty situations | 17 (65.4) | 21 (80.8) |
| Key requirements for disaster preparedness | 10 (38.5) | 21 (80.8) |
| Legal framework for disaster management | 15 (57.7) | 26 (100.0) |
| Recommended percentage of beds for emergencies | 6 (23.1) | 25 (96.2) |
| HEICS in hospital incident management | 10 (38.5) | 25 (96.2) |
| Role of green zone in triage | 6 (23.1) | 13 (50.0) |
| Functional units in the HEICS structure | 3 (11.5) | 14 (53.8) |
| CBRN acronym | 16 (61.5) | 26 (100.0) |
|  |  |  |
| 2. Preparedness and Strategic Planning (PSP) |  |  |
| Challenges for healthcare facilities | 9 (34.6) | 14 (53.8) |
| Four areas of on-site medical facilities | 25 (96.2) | 24 (92.3) |
| PPE selection considerations | 21 (80.8) | 21 (80.8) |
| Key elements of HEICS | 8 (30.8) | 15 (57.7) |
| Focus of reverse triage | 8 (30.8) | 8 (30.8) |
| Indicators for assessment | 10 (38.5) | 23 (88.5) |
| Phases of hospital disaster planning | 6 (23.1) | 13 (50.0) |
| Focus of mitigation phase in disaster management | 2 (7.7) | 18 (69.2) |
| Communication methods for seamless coordination | 17 (65.4) | 26 (100.0) |
| 3. Operational Response and Implementation |  |  |
| Type of triage at the scene of an incident | 19 (73.1) | 21 (80.8) |
| First step in START triage | 6 (23.1) | 18 (69.2) |
| Level C protection usage | 9 (34.6) | 11 (42.3) |
| Purpose of the SAVE triage system | 7 (26.9) | 17 (65.4) |
| Disaster Store location in Emergency Dept | 14 (51) | 14 (51) |
| Triage prioritization post-blast incident | 17 (65.4) | 23 (88.5) |

**- Significance: HEICS: Hospital Emergency Incident Command System; START: Simple Triage and Rapid Treatment; SAVE: Secondary Assessment of Victim Endpoint; CBRN: Chemical, Biological, Radiological and Nuclear*

**Table S3**: *Comparison of Pre- and Post-Training Knowledge and Practice Scores Among Hospital Administrators*

| Questions | Correct answers | |
| --- | --- | --- |
|  | Pre-Test  *n* (%) | Post-Test  *n* (%) |
| **1. Understanding and Awareness (UA)** |  |  |
| - Significance of aligning hospital disaster plans with national guidelines. | 14(53.8) | 15(57.6) |
| - Type of disaster requiring strategic patient influx management. | 25 (96.1) | 26 (100.0) |
| - Primary objective of a hospital disaster preparedness plan. | 13 (50) | 21 (80.7) |
| - Recommended percentage of total beds for hospital treatment capacity, in disaster management plan | 4(15.3) | 24 (92.3) |
| - Indicators for assessing hospital disaster plans. | 5 (9.2) | 14 (53.8) |
| - Key phases in hospital disaster preparedness. | 14(53.8) | 18 (69.2) |
| - Purpose of the HEICS*. | 20 (76.9) | 25 (96.1) |
| - Functional units in the HEICS | 2(7.6) | 19 (73) |
| - Legal framework for disaster management in India. | 19 (73.0) | 26 (100) |
| - Meaning of CBRN* | 18 (69.2) | 25 (96.1) |
| - Phase focusing on post-disaster recovery in the Disaster Management Cycle. | 10 (38.4) | 11(42.3) |
| - Purpose of Job Action Cards in disaster management. | 18 (69.2) | 24 (92.3) |
| - How risk is calculated in disaster management. | 20 (76.9) | 26 (100) |
| **2.** **Preparedness and Strategic Planning (PSP)** |  |  |
| - Importance of regular disaster drills and exercises. | 20 (76.9) | 22 (84.6) |
| - Challenges in managing hospital patient capacity during disasters. | 19 (73.0) | 23 (88.4) |
| - Benefits of a clear chain of command within HEICS. | 14 (53.8) | 20 (76.9) |
| - Importance of secure communication methods in hospitals during a disaster. | 15 (57.6) | 17 (65.3) |
| - Role of public information and media liaison in hospital disaster management. | 23 (88.4) | 26 (100) |
| - Importance of demarcating hospital areas in a disaster management plan. | 15 (57.6) | 22 (84.6) |
| - Role of interdisciplinary coordination in disaster management planning. | 22 (84.6) | 24 (92.3) |
| - Primary focus of disaster preparedness for hospital administrators | 22 (84.6) | 23 (88.4) |
| **3. Operational Response and Implementation (ORI)** |  |  |
| - Key difficulties faced by hospitals post-disaster. | 17 (65.3) | 22 (84.6) |
| - Key requirements for a hospital disaster preparedness plan. | 7 (26.9) | 6 (23.0) |
| - Goal of logistics and supply chain management during mass casualties. | 23 (88.4) | 23 (88.4) |
| - Benefits of working with the media during disasters. | 23 (88.4) | 25 (96.1) |

**- Significance: HEICS: Hospital Emergency Incident Command System; CBRN: Chemical, Biological, Radiological and Nuclear*

**Table S4:** *Comparison of* *Pre- and Post-Training Knowledge and Practice Scores Among Community Frontline Workers*

| Questions | Correct Answers | |
| --- | --- | --- |
|  | Pre-test *n*(%) | Post-test *n*(%) |
| **KNOWLEDGE** |  |  |
| Knowledge of the primary goal of community preparedness | 17(65.38) | 25(96.15) |
| Understanding the importance of drills for frontline workers | 20(76.92) | 23(88.46) |
| Awareness of essential items in a disaster kit | 20(76.92) | 23(88.46) |
| Knowledge of the safest water source during a flood | 23(88.46) | 26(100) |
| Understanding the 30-30 rule in lightning safety | 5(19.23) | 7(26.92) |
| Knowledge of precautions when encountering fallen power lines | 24(92.31) | 26(100) |
| Understanding of increased health risks after a flood | 23(88.46) | 23(88.46) |
| Toxic gas exposure safety | 4(15.38) | 23(88.46) |
| Actions to follow during a flood as per official instructions | 24(92.31) | 26 (100.0) |
| Measures to prevent malaria and dengue after a flood | 22 (84.62) | 22 (84.62) |
| **PRACTICE** |  |  |
| Implementation of the Drop-Cover-Hold technique during an earthquake | 3(11.54) | 25(96.11) |
| Implementation of measures to prevent landslides | 17(65.38) | 18(69.23) |
| Protective actions against smoke inhalation during a forest fire | 11(42.31) | 26 (100) |
| Proper management of cattle during emergencies | 11(42.31) | 20(76.92) |
| Recommended actions when outdoors in a lightning storm | 18 (69.23%) | 26 (100.0%) |
| First aid and treatment for severe lightning injuries | 22 (84.62%) | 23 (88.46%) |
